# Combining CXCL10 gene therapy and radiotherapy improved therapeutic efficacy in cervical cancer HeLa cell xenograft tumor models

**DOI:** 10.3892/ol.2015.3281

**Published:** 2015-05-27

**Authors:** MING ZHAO, QIAN MA, JINHUI XU, SHAOZHI FU, LANLAN CHEN, BIQIONG WANG, JINGBO WU, LINGLIN YANG

**Affiliations:** Department of Oncology, Affliated Hospital of Luzhou Medical College, Luzhou, Sichuan 646000, P.R. China

**Keywords:** CXC chemokine ligand 10, gene therapy, radiotherapy, cervical cancer

## Abstract

Radiotherapy is an important treatment method for cervical cancer, but the efficacy requires improvement. Therefore, novel methods of treatment are required. Previous data have demonstrated that the CXC chemokine ligand 10 (CXCL10) inhibits angiogenesis, induces apoptosis and causes avoidance of the S phase of the cell cycle in cervical cancer cells. The aim of the present study was to evaluate the anti-tumor effect of radiotherapy combined with CXCL10 gene therapy. Mouse models of cervical carcinoma were created by inoculation with HeLa cells, and were treated by combining intravenously administered plasmid-encoding CXCL10, administered 5 times (days 12, 15, 18, 21 and 24 following inoculation), with direct radiation (20 Gy/5 fractions) administered on 5 consecutive days (~day 27 after inoculation). The vessel density and tumor cell proliferation were observed by immunostaining, and apoptosis was determined using a TUNEL assay. The results revealed a significant increase in the inhibition of tumor growth, reduced vessel density, decreased cell proliferation and increased apoptosis in the tumor cells of the combination therapy group. Overall, these findings resulted in the conclusion that CXCL10 gene therapy in combination with radiotherapy is a novel effective therapeutic strategy for cervical cancer.

## Introduction

Cervical cancer is one of the most common cancers worldwide (1) and the leading cause of cancer-associated mortality in women in developing countries (2). The treatment of cervical cancer mainly consists of surgery, chemotherapy and radiotherapy. Radiotherapy is an important therapy for cervical cancer, and it is currently administered to almost 80% of patients with cervical cancer, at various clinical stages (3,4). However, radiotherapy is not extremely effective at treating cervical cancer, particularly in patients at an advanced stage of disease (5,6). Therefore, it is necessary to identify novel methods to combine with radiotherapy to improve the efficacy of treatment.

Chemokines are a superfamily of cytokines that are important regulators of cell migration, lymphocyte recruitment and angiogenesis (7). CXC chemokine ligand 10 (CXCL10), also termed interferon-γ-induced protein 10, is a small cytokine belonging to the CXC chemokine family that was initially characterized as a chemoattractant for activated T lymphocytes. CXCL10 has been identified as a 10-kDa secreted protein induced by interferon-γ (8) in a variety of cell types, including endothelial cells, keratinocytes, fibroblasts, activated monocytes and neutrophils (9). Over previous years, studies have increasingly reported that CXCL10 plays an important role in angiogenesis and tumor growth inhibition (10–12). Our previous study revealed that CXCL10 may induce apoptosis, inhibit angiogenesis and human papillomavirus, resulting in effects against cervical cancer (13). It has also been found that CXCL10 causes the avoidance of the S phase of the cell cycle in cervical cancer cells (14), during which time the cells are least sensitive to radiation.

Therefore, it was assumed that the combination of CXCL10 gene therapy and radiotherapy may improve the inhibition of cervical cancer progression through a variety of mechanisms, to achieve effective coordination in the treatment of cervical cancer.

In the present study, human HeLa cervical carcinoma tumors were established in immunodeﬁcient mice, and CXCL10 gene therapy and radiotherapy were administered to establish the effects on tumor growth.

## Materials and methods

### 

#### Cell lines

The cervical cancer HeLa cell line was obtained from the American Type Culture Collection (Manassas, VA, USA). The cells were cultured in media consisting of Dulbecco's modified Eagle's medium (DMEM) supplemented with 2 mM L-glutamine (GE Healthcare Life Sciences, Logan, UT, USA), plus 100 U/ml penicillin and 100 µg/ml streptomycin (Beyotime Institute of Biotechnology, Haimen, Jiangsu, China), and 10% fetal bovine serum (FBS; GE Healthcare Life Sciences).

#### Plasmids

A DNA fragment encoding CXCL10 was cloned into the pcDNA3.1 vector (Invitrogen, Carlsbad, CA, USA) between the restriction sites for *Eco*RI and *Xho*I. The plasmid was puriﬁed by two rounds of passaging over Endo-free columns (Qiagen, Valencia, CA, USA), as previously described (15).

#### Reverse transcription-quantitative polymerase chain reaction (PCR)

Total RNA was isolated from HeLa cells transfected with the pcDNA3.1-CXCL10 plasmid using TRIzol reagent (Invitrogen), according to the manufacturer's instructions. Total RNA was reverse transcribed to cDNA using the reverse transcription system from Promega (Madison, WI, USA). PCR was subsequently performed with an initial denaturation step that was performed at 94°C for 4 min, followed by 28 cycles at 94°C for 20 s, 60°C for 20 s and 72°C for 30 s, with a final extension of 72°C for 4 min. The primers used for PCR were as follows: CXCL10 forward, 5′-CCTTATCTTTCTGACTCT AAGTGGC-3′ and reverse, 5′-ACGTGGACAAAATTG GCTTG-3′; GAPDH forward, 5′-TCATCTCTGCCCCCT CTG-3′ and reverse, 5′-CCTGCTTCACCACCTTCTTG-3′.

#### Tumor formation in nude mice

To establish the cervical cancer tumors, eight-week-old female nude mice (Beijing HFK Bioscience Co., Ltd, Beijing, China) were inoculated with 1×10^6^ HeLa cells subcutaneously in the right flank. Tumor dimensions were measured with calipers every 3 days, and tumor volumes were calculated as follows (16):

Tumor volume (mm^3^) = 0.5 × length × width^2^

Animal experiments were conducted according to institutional guidelines concerning animal use and care, and the study was approved by the ethics committee of Luzhou Medical College, (Luzhou, China).

#### CXCL10 gene therapy

The mice were randomly divided into five groups when the tumors were ~15 mm^3^ in volume, which occurred ~12 days subsequent to HeLa cell inoculation, and each group consisted of 10 mice. The recombinant CXCL10 or pcDNA3.1 plasmids were gently combined with a liposomal transfection agent in a 1:3 ratio (50:150 µg for each mouse), and incubated at room temperature for ~30 min. The mixture was then administered intravenously 5 times on days 12, 15, 18, 21 and 24. Mice from the phosphate-buffered saline (PBS) group were injected with PBS as the control agent and acted as the control group.

#### Radiotherapy

Radiotherapy was performed when the tumors were ~300 mm^3^ in volume, which occurred ~27 days subsequent to inoculation of the mice with HeLa cells. The tumors were radiated with 6-MV X-rays, and each mouse was administered with a total radiation dose of 20 Gy, with 4 Gy being administered for each dose on 5 days, at the rate of 200 cGy/min.

#### Histological analysis

The mice were sacrificed 39 days subsequent to inoculation with the HeLa cells and the tumors were removed and embedded in parafﬁn. Tissue sections were immunostained with rat anti-mouse monoclonal antibody against cluster of differentiation 31 (CD31; catalog no. 553369; BD Biosciences, Franklin Lakes, NJ, USA; dilution, 1:200) or rabbit anti-human polyclonal antibody against Ki-67 (catalog no. 19972-1-AP; Proteintech Group, Inc., Wuhan, Hubei, China; dilution, 1:100). Vessel density was calculated by counting the number of microvessels per high-power ﬁeld (magnification, x200; Leica DM3000, Leica Microsystems GmbH, Wetzlar, Germany) in the tumor sections, as previously described (17). The rate of cell proliferation was calculated for five random fields by dividing the number of Ki-67-positive cells by the total number of cells. TUNEL was performed using an *in situ* cell-death detection kit (Roche, Mannheim, Germany). In total, five ﬁelds were randomly selected and analyzed. The apoptotic index was calculated as the ratio of the apoptotic cell number to the total tumor cell number in each ﬁeld.

#### Statistical analysis

The data were assessed by a one-way analysis of variance and Student's *t*-test, and a post-hoc test was conducted according to the Student-Newman-Keuls method. P<0.05 was considered to indicate a statistically significant difference.

## Results

### 

#### Overexpression of CXCL10 in HeLa cells

The recombinant plasmid encoding CXCL10 was cloned, and the transcript of CXCL10 mRNA was confirmed in transfected cells, as revealed by RT-PCR.

#### Combination of CXCL10 gene therapy and radiotherapy markedly inhibits cervical cancer tumor growth

**Figure 2.**
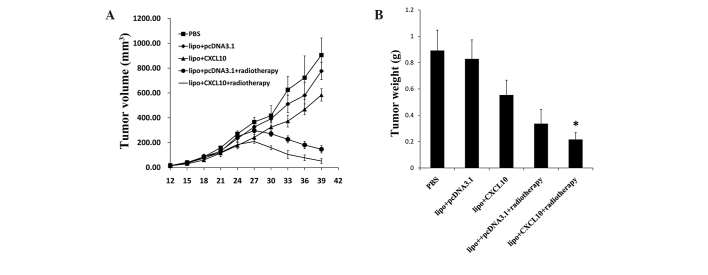
CXCL10 gene therapy combined with radiotherapy markedly inhibited the growth of cervical cancer tumors. The (A) volume and (B) weight of tumors treated with PBS, lipo + pcDNA3.1, lipo + CXCL10, lipo + pcDNA3.1+radiotherapy or lipo + CXCL10 + radiotherapy was quantitated. Error bars express the standard error of the entire treatment group. *P<0.05. CXCL10, CXC chemokine ligand 10; PBS, phosphate buffered saline; lipo, liposomal transfection agent.

The effects of CXCL10 gene therapy and radiotherapy alone and in combination on xenograft tumor models of cervical cancer were assessed in nude mice. The tumor volumes were measured every 3 days subsequent to inoculation with HeLa cells. The tumor volume in the PBS and pcDNA3.1 control groups increased rapidly. By contrast, the tumor growth rate in nude mice treated with CXCL10 gene therapy alone decreased compared with the control group, and this decline was more evident in the group treated with radiotherapy. In addition, the combined treatment of CXCL10 gene therapy and radiotherapy inhibited tumor growth more effectively compared with all other groups (P<0.05) (Fig. 2A). Similarly, the treatment of tumors demonstrated corresponding effects on tumor weight (Fig. 2B). Overall, CXCL10 gene therapy in combination with radiotherapy significantly inhibited cervical cancer tumor growth.

#### Effect of CXCL10 gene therapy and radiotherapy on angiogenesis in cervical cancer

**Figure 3.**
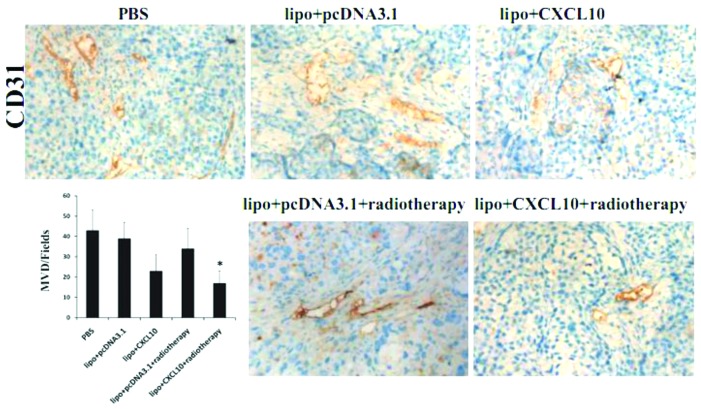
Effect of CXCL10 gene therapy and radiotherapy on angiogenesis in cervical cancer. The vascular structures in tumors treated with PBS, lipo + pcDNA3.1, lipo + CXCL10, lipo + pcDNA3.1 + radiotherapy or lipo + CXCL10 + radiotherapy, were detected by immunohistochemical staining for CD31. The combination group, which was treated with CXCL10 gene therapy and radiotherapy, exhibited a significantly decreased vessel density compared with any other groups. Error bars represent standard error of the entire treatment group. *P<0.05 vs. controls. CXCL10, CXC chemokine ligand 10; PBS, phosphate buffered saline; CD31, cluster of differentiation 31; lipo, liposomal transfection agent.

Immunohistochemical labeling of microvessels by CD31 in tumor sections revealed a decrease in tumor vessel density in the CXCL10 group and radiotherapy group compared with those in the control group. In combination, treatment with CXCL10 plus radiotherapy appeared more effective at reducing microvessel density compared with the other groups (P<0.05; Fig. 3).

#### Effect of CXCL10 gene therapy and radiotherapy on cell proliferation in cervical cancer

**Figure 4.**
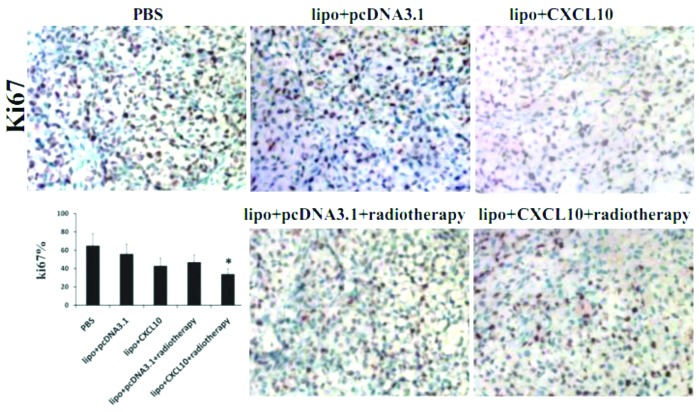
Effect of CXCL10 gene therapy and radiotherapy on cell proliferation in cervical cancer cells. Tissue specimens were obtained from the tumors treated with PBS, lipo + pcDNA3.1, lipo + CXCL10, lipo + pcDNA3.1 + radiotherapy and lipo + CXCL10+radiotherapy. The proliferation rate of tumor cells was determined by Ki-67 staining. The Ki-67 positive cells divided by the total number of cells was evidently decreased in the combination group. Error bars represent the standard error of the entire treatment group. *P<0.05 vs. controls. CXCL10, CXC chemokine ligand 10 PBS, phosphate buffered saline; lipo, liposomal transfection agent.

Cell proliferation was analyzed by immunohistochemical staining of Ki-67 in tumor sections. The number of Ki-67-positive cells were calculated and divided by the total number of cells. A slight but significant decrease in the number of Ki-67-positive cells was observed in the CXCL10 and radiotherapy groups compared with the control group (P<0.05). Additionally, a greater reduction in the number of Ki-67-positive cells was identified in the combination group (P<0.05; Fig. 4).

#### Effect of CXCL10 gene therapy and radiotherapy on cell apoptosis in cervical cancer

**Figure 5.**
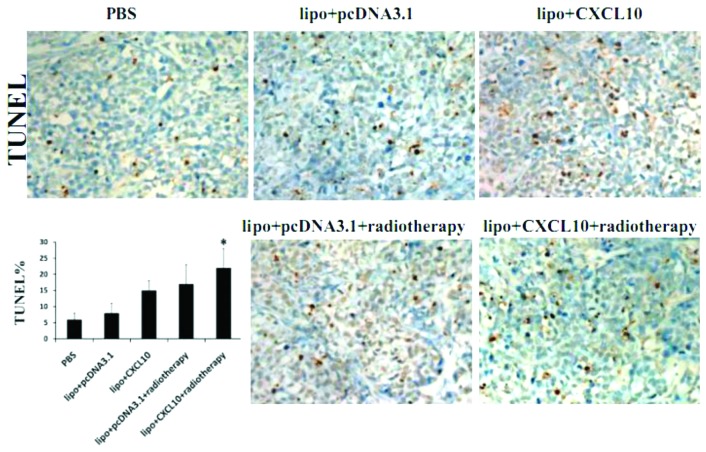
Effect of CXCL10 gene therapy and radiotherapy on cell apoptosis in cervical cancer cells. The apoptotic index was detected by TUNEL assay. The combination group, which was treated with CXCL10 gene therapy and radiotherapy, demonstrated a significant increase in apoptosis compared with the other treatment groups. Error bars express the standard error of the entire treatment group. *P<0.05 vs. controls. CXCL10, CXC chemokine ligand 10; PBS, phosphate buffered saline; lipo, liposomal transfection agent.

The apoptotic rate was analyzed by TUNEL assay in the five groups. As shown in Fig. 5, the cellular apoptosis rate in the CXCL10 group and radiotherapy group was found to be ~10% higher compared with the control, while the rate was even higher in the combination group compared with the CXCL10 and radiotherapy groups (P<0.05) (Fig. 5).

## Discussion

Previously, CXCL10 has been demonstrated to be involved in cell cycle regulation in a podocyte cell line, with increased p27^Kip1^ expression and decreased cyclin E expression (18). Similar results were obtained using HeLa cells in previous studies (17). p27^Kip1^ and cyclin E are crucial cell cycle regulators that are involved in arresting the cell cycle at the G1 phase (19) and promoting cell cycle progression to the S phase (20), respectively. Reduced cyclin E expression prevents cell cycle transition from the G1 to S phase, so the cells remain in the G1 phase. Therefore, CXCL10 may inhibit cell proliferation by influencing cell cycle-associated proteins.

It has been demonstrated that radiation destructs genome DNA to induce cell apoptosis (21); however, S phase, during which time gene synthesis is active in tumor cells, is least sensitive to radiation (22). Therefore, avoidance of the S phase in cancer cells is important for the improvement of the efficacy of radiotherapy. On the basis of our previous analysis (14), it was assumed that CXCL10 is a crucial factor that arrests the cell cycle at G1 phase to avoid the S phase, and therefore promotes the efficacy of radiotherapy.

In the present study, treatment with liposome-encapsulated CXCL10 gene therapy and radiotherapy was combined to assess the resulting inhibition of growth in cervical cancer tumors. The present results revealed that CXCL10 gene therapy and radiotherapy each attenuated tumor growth when administered separately. In addition, the combination of the two therapies demonstrated a significantly increased inhibitory effect on tumor growth. To understand the mechanism of the growth inhibition, tumor sections were analyzed for angiogenesis, cell proliferation and apoptosis. It was found that CXCL10 gene therapy combined with radiotherapy enhances the reduction of microvessel density, decrease in cell proliferation and upregulation of apoptosis compared with each treatment alone.

Overall, the present study indicated that the combination of CXCL10 gene therapy and radiotherapy is an efficient strategy for growth suppression in cervical tumors, which may result from the attenuation of angiogenesis, decline in cell proliferation and induction of apoptosis. This approach is novel as it hypothesizes CXCL10 may enhance the radiosensitivity of cervical tumors in nude mice. Since the present protocol was not performed using any other tumor model, whether this approach may be applied to other tumor types remains unknown, and additional investigations are required.

## Figures and Tables

**Figure 1.**
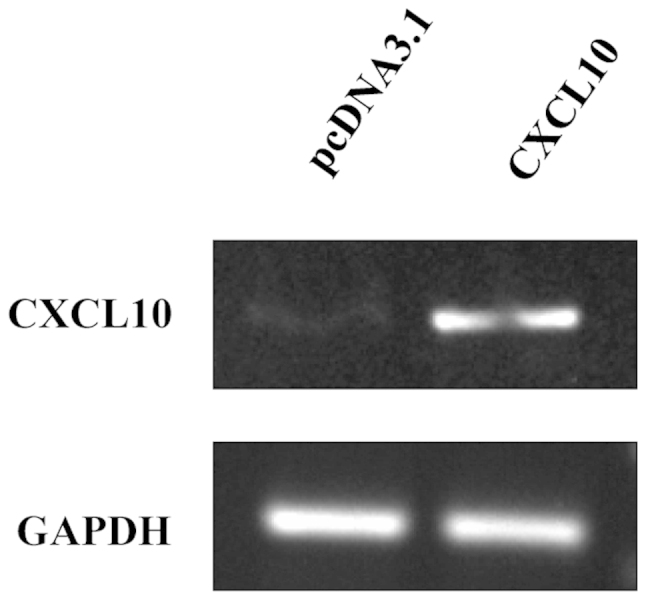
Detection of the CXCL10 mRNA transcript in HeLa cells. CXCL10 mRNA was clearly enhanced in transfected HeLa cells, as determined by reverse transcription-quantitative polymerase chain reaction. CXCL10, CXC chemokine ligand 10.
